# Evaluation of leflunomide for the treatment of BK viremia and biopsy proven BK nephropathy; a single center experience

**DOI:** 10.15171/jnp.2016.06

**Published:** 2015-12-23

**Authors:** Nicole Nesselhauf, Jaclyn Strutt, Bahar Bastani

**Affiliations:** ^1^Department of Pharmacy, SSM Saint Louis University Hospital, Saint Louis, Missouri, USA; ^2^Department of Pharmacy, Minneapolis VA Health Care System, Minneapolis, Minnesota, USA; ^3^Division of Nephrology, Saint Louis University, School of Medicine, Saint Louis, Missouri, USA

**Keywords:** BK virus, Polyomavirus, BK viremia, BK nephropathy, Kidney transplant, Leflunomide

## Abstract

*Background:* BK virus reactivation is a significant complication following renal transplantation that can result in graft failure. Reduction of immunosuppression and substitution of leflunomide for mycophenolate mofetil (MMF) has been used to treat this entity.

*Objectives:* To evaluate the use of leflunomide in BK viremia (BKV) and biopsy proven BK nephropathy (BKN) in kidney and kidney-pancreas transplant recipients.

*Patients and Methods:* We retrospectively reviewed 28 kidney and kidney-pancreas transplant recipients who had received leflunomide for BKV from January 2006 to November 2012. Demographics, time to BKV diagnosis, biopsy findings, rejection episodes, and laboratory data were recorded.

*Results:* The average (mean ± SD) time to BKV from time of transplant was 316.1 ± 368.0 days (62-1708 days). At time of diagnosis, 64% of patients had their maintenance immunosuppression reduced. The indications for leflunomide administration were; BKV and biopsy proven acute rejection (BPAR) (50%), biopsy proven BKN (18%), or persistent BKV (25%). Therapeutic levels (50-100 mcg/mL) were achieved in only 54% of patients, and 60% of them had required a leflunomide dose of at least 60 mg/day. BK virus was cleared from the serum on average of 151 ± 145.2 days (17-476 days). At study commencement, 29% of patients had remained on leflunomide due to persistent BKV.

*Conclusions:* In our study, most patients required at least a 60 mg daily dose of leflunomide to achieve therapeutic levels and to clear the virus compared to the standard 40 mg daily dose. Delaying therapy may result in progressive BKV and BKN.

**Implication for health policy/practice/research/medical education:**

The use of more potent immunosuppressive medications in transplantation has decreased the rate of rejection; however, it has resulted in increased incidence of BK viremia (BKV), BK virus associated nephropathy, and subsequent graft failure. Decreasing total immune suppression and substituting leflunomide for mycophenolate has been used to remedy cases of BK nephropathy (BKN) and BKV with concomitant rejection. We present our experience with the use of leflunomide in such situations.

## 1. Background

BK virus associated nephropathy (BKN) is a significant complication following renal transplantation that can result in graft failure. BK virus is a human polyomavirus that is widespread in the general population. It is estimated that greater than 90% of the population become seropositive for BK virus within the first decade of life (1). BK virus is colonized in the epithelial cells of renal medulla and urinary tract. It can reactivate in immunocompromised patients and can lead to nephropathy and graft failure (1). BKN is estimated to occur in 5%-10% of renal transplant recipients with reports of graft loss in 10%-80% of these patients (2). The standard of care for treatment of BK viremia (BKV) and BKN is reduction in immunosuppressant medications (1). Calcineurin inhibitors can significantly inhibit BK virus specific T-cells. Egli and colleges found that tacrolimus concentrations above 6 ng/ml inhibited BK virus specific T-cells by 50%. However, when tacrolimus levels were reduced to below 3 ng/ml the inhibition was less than 30%. Similar results were found with cyclosporine; 50% reduction in BK virus specific T-cells with cyclosporine level of 1920 ng/ml, and 30% reduction with levels less than 960 ng/ml (3). Therefore, immunosuppression reduction, specifically with calcineurin inhibitors is recommended as first line therapy for treating BKV (1-3). However, despite immunosuppression reduction, some patients continue to experience persistent BKV or BKN. Several adjuvant therapies, such as leflunomide, cidofovir, intravenous immunoglobulin (IVIG), and ciprofloxacin have been tried; however, guidelines for their use are not well established. These therapies have shown varying results in their effectiveness to lower BK viral loads and prevent or treat BKN. Leflunomide has been utilized because it displays both antiviral and immunosuppressive properties. Leflunomide is an anti-metabolite, antirheumatic disease modifying agent. It is approved for the treatment of rheumatoid arthritis. Leflunomide inhibits pyrimidine synthesis resulting in anti-proliferative and anti-inflammatory effects. Its metabolite, teriflunomide (A77 1726) has been found to reduce or stop the replication of BK in vitro and in animal models (2,4). Leflunomide’s mechanism of action in reducing BK viral load is unknown; it is thought to be similar to its effects on CMV, i.e., decreasing viral replication by disruption of viron assembly at the nucleocapsid.

## 2. Objectives

We sought to evaluate the use of leflunomide in BKV and biopsy proven BKN in kidney and kidney-pancreas transplant recipients.

## 3. Patients and Methods

We routinely screen all post renal transplant patients with monthly serum BK virus polymerase chain reaction (PCR) for the first year and every 3 months for the subsequent 2 years. The first line treatment for BKV at our institution is immunosuppression reduction. The patients’ mycophenolate mofetil (MMF) dose is decreased by 50% and the target tacrolimus level may also be reduced. At our institution we reserve leflunomide for patients with biopsy proven BKN, BKV with concomitant biopsy proven acute rejection (BPAR), or BKV with elevated or rising donor specific antibodies. Leflunomide is started with a loading dose of 100 mg daily for 5 days followed by 40 mg daily. Monthly plasma levels are followed with a goal of 50-100 mcg/ml MMF is discontinued after leflunomide has been started. Once the patient has cleared the virus, leflunomide is discontinued and the patient resumes a lower dose of MMF. The objective of this study was to retrospectively evaluate the use of leflunomide in BKV and biopsy proven BKN at Saint Louis University Hospital.

We retrospectively reviewed 28 kidney and kidney-pancreas transplant patients that received leflunomide for BKV from January 2006 to November 2012 at Saint Louis University Hospital. All patients had received antibody induction per transplant center protocol, and had received maintenance immunosuppression with tacrolimus and MMF, with or without prednisone. In patients who were transplanted between 2006 and 2009, alemtuzumab 30 mg as a single dose with a rapid methylprednisolone taper was given for induction therapy. From 2009 to present, rabbit antithymocyte globulin (rATG) 2 mg/kg daily for three doses with tapering methylprednisolone was the induction protocol. Basiliximab 20 mg on day 0 and day 4 was given in place of thymoglobulin when patients were deemed very low risk for transplant rejection. Patients who had received alemtuzumab did not receive maintenance steroids, while patients who had received rATG or basiliximab were maintained on prednisone 5 mg daily from the fifth post-operative day. Saint Louis University Hospital is a 350 bed academic teaching hospital. The following data were collected and analyzed: time from transplant to BKV diagnosis, serum BKV PCR, urine BK virus PCR, time to BKV clearance, leflunomide indication, leflunomide initial loading and maintenance dose, leflunomide dose to achieve therapeutic level, time to achieve therapeutic level of leflunomide, duration of leflunomide therapy, side effects from leflunomide, rejection after BKV, BKV relapse, return to dialysis, and death.

### 3.1. Ethical issues

The research protocol was approved by the IRB committee of Saint Louis University School of Medicine.

### 3.2. Statistical analysis

Data was expressed using descriptive statistics.

## 4. Results

The transplant center preformed approximately 57 kidney transplants per year during the study period. The transplanted population is primarily Caucasian and African American. The baseline characteristics are listed in Table 1. The majority of the study population were male. Most patients received induction therapy with rATG. The average (mean ± SD) time from transplant to diagnosis of BKV was 316.1 ± 368 days (62-1708 days). At the time of diagnosis, 64% of patients had their maintenance immunosuppression reduced by decreasing tacrolimus goal and reduction of MMF. Indications for using leflunomide are shown in Table 2. Leflunomide was started in patients with BKV and concomitant BPAR (50%), BKN (18%), BKV and DSA (7%), and persistent BKV (25%). Loading doses of leflunomide were given to most patients (89%). The most common loading dose was 100 mg daily for 5 days; however, a few patients received 100 mg daily for 3 days. Therapeutic levels (50-100 mcg/mL) were achieved in 15 patients (54%). In those patients who achieved therapeutic levels, 61% had required a leflunomide dose of at least 60 mg/day. The mean time to therapeutic level was 60.9 ± 33.2 days (39-137 days). BK virus was cleared from the serum on average of 151 ± 145.2 days (17-476 days). The maintenance dose required to clear the virus varied between patients (Table 3). The median dose required to clear the virus was 60 mg per day (n = 20). Seventy-one present of the patients cleared their BKV, while 21% never cleared their viremia. At study commencement, 29% of patients remained on leflunomide, for an average duration of 517 ± 547 days (72-1719 days), due to persistent BKV or as maintenance immunosuppression.

**Table 1:** Baseline characteristics of 28 patients who received leflunomide

** Mean age (y) ± SD**	**51.9 ± 13.1**
Male, n (%)	25 (89)
Transplant type, n (%)	
Kidney	26 (93)
Kidney –Pancreas	2 (7)
Induction immunosuppression therapy , n (%
Antithymocyte globulin (rabbit))	16 (57)
Alemtuzumab	8 (29)
Basiliximab	1 (3)
Unknown induction	3 (11)
Maintenance immunosuppression therapy, n (%	
Tacrolimus, MMF, prednisone	20 (71)
Tacrolimus, MMF	5 (18)
Tacrolimus, prednisone	2 (7)
Tacrolimus alone	1 (4)
Creatinine at diagnosis, (mg/dL); mean (IQR), n (%)	2 (1.5-2.4)
Tacrolimus level at diagnosis, (ng/mL); mean (IQR), n (%)	8.7 (5.5-10.4)

Abbreviation: IQR, interquartile range.

**Table 2:** Indications for the use of leflunomide (n = 28)

** BKV with concomitant BPAR, n (%)**	**14 (50)**
BKN, n (%)	5 (18)
BKV and Donor Specific Antibody(s), n (%)	2 (7)
Persistent BKV, n (%)	7 (25)

Abbreviations: BKV, BK viremia; BPAR, biopsy proven acute rejection; BKN, biopsy proven BK nephropathy.

**Table 3:** Daily leflunomide dose required to clear BKV (n = 28)

** 20 mg, n (%)**	**2 (7)**
40 mg, n (%)	7 (25)
60 mg, n (%)	9 (32)
80 mg, n (%)	2 (7)
Never cleared BKV, n (%)	6 (21)
Lost to follow-up, n (%)	2 (7)

Abbreviation: BKV, BK viremia.

Patient complications are shown in Table 4. During leflunomide treatment, 11 patients (39%) developed complications. Of these eleven: 10 patients experienced leukopenia, 3 thrombocytopenia, 1 hepatotoxicity, and 3 anemia. While receiving leflunomide, 6 patients experienced BPAR, 4 of who resulted in graft failure and returned to dialysis. Two other patients also returned to dialysis, and 2 died during the study period.

**Table 4:** Patients with complications (n = 11)

Leflunomide Side effects, n (%)	
Leukopenia	10 (36)
Thrombocytopenia	3 (11)
Anemia	3 (11)
Hepatotoxicity	1 (4)
Rejections, n (%)	6 (21)
Return to dialysis, n (%)	6 (21)
Death, n (%)	2 (7)

## 5. Discussion

BKV is a significant problem in renal transplant recipients. There is no treatment guidelines published for BKV and risk factors are also not well established. It is known that BKV occurs more often in kidney transplant recipients than any other solid organ or bone marrow and stem cell transplants. It is thought that male gender may be a risk factor for developing BKV (1). In our study population, 89% of patients were male. In 2007, Faguer and colleagues prospectively followed 12 kidney transplant patients who had developed BKN and were treated with leflunomide. They found that clearance of BKV was achieved in 42% of cases, and 17% of patients experienced adverse effects (5). In the present study 71% of patients receiving leflunomide with immunosuppression reduction, for indications shown in Table 2, cleared the virus, however, 39% of patients experience side effects.

Appropriate leflunomide dosing and consistent monitoring are important to achieving therapeutic levels and viral clearance. Hirsch and colleagues recommend a leflunomide loading dose of 100 mg daily for 5 days followed by an initial maintenance dose of 40 mg daily (1). However, we found that 60% of patients required a leflunomide dose of at least 60 mg/day to achieve therapeutic levels (50-100 mcg/mL) and to clear the BKV. Williams and colleagues found a statistically significant difference in viral clearance or progressive reduction in the viral load in blood and urine when patients had blood levels greater than 40 mcg/mL compared to less than 40 mcg/mL (6). In order to achieve therapeutic levels, we propose to start leflunomide 100 mg daily for 5 days followed by 60 mg daily as maintenance. Leflunomide levels should be monitoring every month until therapeutic levels are achieved.

## 6. Conclusions

Transplant centers should develop and follow a protocol for BKV surveillance, treatment, and monitoring. From this study it is reasonable to conclude that patients receiving leflunomide should be loaded 100 mg daily for 5 days followed by a maintenance dose of 60 mg daily instead of the previously suggested 40 mg daily dose.

## 7. Limitations of the study

We recognize there are several limitations to our study. Our study design was retrospective and looked at a small population of patients. There were several confounding variables when looking at transplant patients, such as confounding medications and disease states. Leflunomide side effects did not take into consideration other medications patients were concurrently taking.

## Authors’ contribution

All authors participated in design, performance, and preparation of the manuscript.

## Conflicts of interest

The authors declared no competing interests.

## Funding/Support

None.
